# Efficacy of ^99m^Tc-DTPA SPECT/CT in diagnosing Orbitopathy in graves’ disease

**DOI:** 10.1186/s12902-019-0340-0

**Published:** 2019-01-18

**Authors:** Piotr Szumowski, Saeid Abdelrazek, Łukasz Żukowski, Małgorzata Mojsak, Monika Sykała, Katarzyna Siewko, Katarzyna Maliszewska, Anna Popławska-Kita, Janusz Myśliwiec

**Affiliations:** 1 Department of Nuclear Medicine, Medical University of Bialystok, M. Skłodowskiej-Curie St. 24A, 15–276 Bialystok, Poland; 2 Department of Endocrinology, Diabetology and Internal Medicine, Medical University of Bialystok, M. Skłodowskiej-Curie St. 24A, 15–276 Bialystok, Poland

**Keywords:** ^99m^Tc-DTPA, Graves’ orbitopathy, SPECT/CT, MRI

## Abstract

**Background:**

The most frequently used methods of assessing Graves’ orbithopathy (GO) include: Clinical Activity Score (CAS), ultrasonography (USG), computed tomography (CT), and magnetic resonance imaging (MRI). There exists another, slightly forgotten, imaging method: single-photon emission computed tomography (SPECT) with the use of diethylenetriaminepentaacetic acid tagged with ^99m^Tc (^99m^Tc-DTPA). These days it is possible to conduct a SPECT examination fused with a CT scan (SPECT/CT), which increases the diagnostic value of the investigation. The aim of this paper is to evaluate the usefulness of ^99m^Tc-DTPA SPECT/CT in diagnosing Graves orbitopathy, as compared with other methods.

**Methods:**

Twenty-three patients with suspected active (infiltrative-edematous) Graves’ orbithopathy were included in the study. Each patient underwent a CAS, an MRI, and a SPECT/CT. The obtained results were analysed statistically, with the assumed statistical significance of *p* < 0.05.

**Results:**

The SPECT/CT and MRI were found to have the highest sensitivity: 0.93 each. The SPECT/CT had the highest specificity: 0.89. MRI and CAS had lower values: 0.78 and 0.56, respectively. The occurrence of an active form of GO had no impact on the exacerbation of exophthalmos or the thickness of the oculomotor muscles.

**Conclusions:**

The ^99m^Tc-DTPA SPECT/CT method provides a very good tool for assessing the active form of GO and can, alongside the MRI scan, be used as a referential diagnostic procedure in GO.

## Background

Graves’ orbitopathy (GO) is a complex of ocular symptoms caused by autoimmune inflammation of the soft tissue in the orbital cavity. Active inflammation of the orbital cavity’s soft tissue initially presents only with swelling, but in the long term can lead to irreversible lesions in the form of fibrosis and steatosis [1].

Because the condition can resolve spontaneously, the decision to administer treatment should be preceded by an assessment of the intensity of inflammation. The most frequently used methods of evaluating Graves’ orbithopathy include: Clinical Activity Score (CAS), ultrasonography (USG), computed tomography (CT), and magnetic resonance imaging (MRI). There exists another, slightly forgotten, imaging method: single-photon emission computed tomography (SPECT) with the use of diethylenetriaminepentaacetic acid tagged with ^99m^ Tc (^99m^Tc-DTPA). The investigation involves intravenous (iv) administration of the ^99m^Tc-DTPA radiotracer (used mainly in testing renal clearance), which then becomes extravasated at the site of inflammation, to combine with the polypeptides of the extracellular inflammatory fluid. The volume of ^99m^Tc-DTPA’s accumulation at the site of inflammation, in this case in the soft tissue of the orbital cavity (mainly in the oculomotor muscles) is directly proportionate to the activity of the infiltrative and edematous process [2]. The method employs a gamma camera to capture gamma radiation produced by the ^99m^Tc attached to DTPA, using the single-photon emission computed tomography (SPECT) technique. Modern gamma cameras are hybrid devices, combined with CT, which make it possible to conduct scintigraphy together with computed tomography (SPECT/CT), thus increasing the diagnostic accuracy of the test.

In view of the above, the purpose of this paper is to assess the efficacy of SPECT/CT scintigraphy using ^99m^Tc-DTPA in diagnosing Graves’ orbitopathy, in comparison with other methods.

## Methods

### Patients

Twenty-three patients (women aged 25–60, 45 on average) with suspected active (infiltrative-edematous) Graves’ exophthalmos were included in the study. All the patients had been diagnosed with Graves’ disease, confirmed by the presence of above normal (norm: < 2 IU/l) levels of TRAb receptor antibodies in the blood serum. Fifteen of them were on antithyroid drugs in order to normalise serum levels of the thyroid hormones. The others were hypothyroid, post-radioiodine treatment and on thyroxine substitution therapy. The evaluation of GO involve using: the CAS score, the MRI test, and the SPECT/CT imaging technique. Each patient had these tests performed within 10 days after the commencement of the diagnostic process.

### CAS score

The seven-point scale used for evaluating the clinical activity of GO is recommended by the European Group on Graves’ Orbitopathy (EUGOGO). It takes into account the following clinical symptoms, each of which represents one point on the scale: retroorbital pain, pain with eye movement, swelling of the eyelids, redness of the eyelids, conjunctival redness, conjunctival swelling, inflammation of the caruncle or plica. The presence of at least 3 out of the 7 above-mentioned clinical symptoms indicates an active form of GO [3].

### MRI

The MRI of the orbital cavities was performed by means of a 3 T MRI scanner (Biograph mMR, Siemens, Germany), using the T1 sequence with and without intravenous contrast (Gadolin), as well as the T2 sequence. All the scans were conducted in the coronal and axial planes, with a slice thickness of 3 mm. The presence of infiltration and oedema within oculomotor muscles (T2 signal increased and T1 signal strengthened in inflamed muscles on contrast-enhanced scan) was taken as a positive result, whereas the absence of the active form of GO was considered to be a negative result [4].

### SPECT/CT

**Fig. 1:**
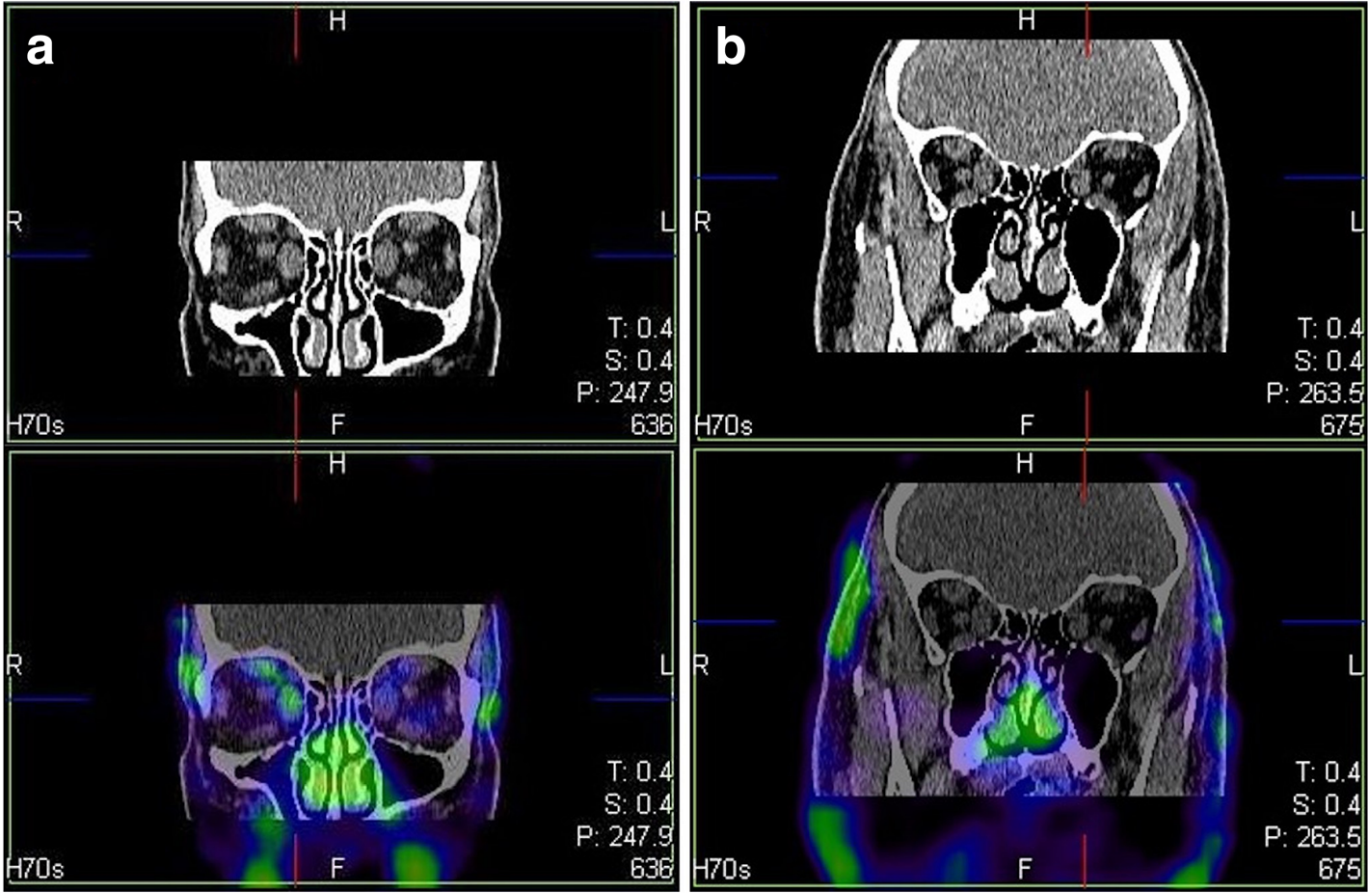
^99m^Tc-DTPA SPET/CT scintigraphy of the orbital cavity in coronal planes. All the rectus muscles of the eye are thickened: **a**) with increased accumulation of ^99m^Tc-DTPA only in the medial and superior muscles of the right eye, which is indicative of active GO in the medial and superior muscles. **b** without increased accumulation of ^99m^Tc-DTPA, which is indicative of non-active GO. The physiological 99mTc-DTPA uptake of nasal mucosa is visible at both cases. Abbreviations: MRI = magnetic resonance imaging; SPECT-CT = fused single photon emission computed tomography with computed tomography; GO = Graves’ orbitopathy; ^99m^Tc-DTPA = diethylenetriaminepentaacetic acid tagged with ^99m^Tc

The SPECT/CT of orbital cavities was conducted with the use of the radiotracer ^99m^Tc-DTPA (PolAtom, Polska), given intravenously at the dose of 7 MBq/kg. The 3D tomography using a SPECT/CT gamma camera (Symbia T, Siemens, Germany) was performed at 20 min after radiotracer administration. The SPECT image was obtained through the application of iterative reconstruction. The voltage of the RTG lamp used for the scans equalled 80 kV and the anodic current - 30 mA. Then, the fused SPECT and CT (SPECT/CT) images with a slice thickness of 1.25 mm were analysed medically. Increased accumulation of the radiotracer in the muscles or in the oculomotor muscles, as compared with the background (i.e. the uptake of the radiotracer in the remaining craniofacial muscles) were taken as a positive result, i.e. presence of infiltrative-edematous lesions (Fig. 1).

As part of the SPECT/CT examination, an additional assessment was made of the thickness of the oculomotor muscles and the degree of the exophthalmos. The evaluation of the exophthalmos was performed in the standard manner on the basis of CT scans in the axial plane, obtained as a result of the SPECT/CT. The straight line joining the anterior tips of the zygomatic bones was the line of reference. The distance of > 23 mm from the line of reference to the front border of the eye was interpreted as indicative of exophthalmos. The thickness of the oculomotor muscles was measured using the scans obtained as a result of the SPECT/CT examination [5].

### Statistical analysis

The statistical analysis of the study results was performed using Statistica 13.1 software (Stat Soft, Tulsa, USA).

To assess the usefulness of the diagnostic methods (CAS, MRI, SPECT/CT) in detecting the active form of GO, contingency tables were created, which included such measures as: sensitivity, specificity, positive predictive value (PPV), negative predictive value (NPV), and accuracy (ACC).

The coefficient of confidence for each measure was calculated on the basis of the Clopper-Pearson method for an individual proportion and equalled 0.95.

The evaluation of the dependence between the degree of exophtalmos and the overall thickness of the oculomotor muscles and the presence of an active inflammatory form of GO, as well as the comparison of the studied methods (CAS, MRI, SPECT/CT) was conducted by means of one-way analysis of variance.

## Results

When analysing the results of CAS, MRI, and SPECT-CT in detecting an active form of GO in the examined patients, one should bear in mind that the diagnoses were not always consistent (Table 1). Therefore, the following assumptions were adopted in order to perform the statistical analysis:

- result of the test was taken as truly positive (active form of GO) if at least two of the compared methods yielded positive results;
- result of the test was taken as truly negative (non-active form of GO) if at least two of the compared methods yielded negative results;
- result of the test was taken as falsely negative if only one of the compared methods yielded a negative result;
- result of the test was taken as falsely positive if only one of the compared methods yielded a positive result;

**Table 1:** Results of active (+) and non-active (−) forms of GO in 23 patients after CAS, MRI, and SPECT/CT

	GO patients																						
	1	2	3	4	5	6	7	8	9	10	11	12	13	14	15	16	17	18	19	20	21	22	23
CAS	**–**	**+**	**–**	**+**	**+**	**+**	**+**	**+**	**+**	**+**	**+**	**+**	**+**	**+**	**+**	**+**	**+**	**+**	**–**	**–**	**–**	**–**	**–**
MRI	**+**	**+**	**+**	**–**	**+**	**+**	**–**	**–**	**+**	**+**	**–**	**–**	**–**	**+**	**+**	**+**	**+**	**+**	**–**	**+**	**+**	**+**	**–**
SPECT/CT	**–**	**–**	**+**	**+**	**+**	**+**	**–**	**–**	**+**	**+**	**–**	**–**	**–**	**+**	**+**	**+**	**+**	**+**	**–**	**–**	**+**	**+**	**+**

Abbreviations: +, positive result (active GO); −, negative result (non-active GO); *CAS* clinical activity score, *MRI* magnetic resonance imaging, *SPECT-CT* fused single photon emission computed tomography with computed tomography, *GO* Graves’ orbitopathy

Next, taking into consideration the above assumptions, contingency tables were constructed for the particular diagnostic methods: CAS, MRI, and SPECT/CT (Tables 2, 3, and 4).

**Table 2:** Correlation between real occurrence of active or non-active GO and result of CAS (contingency table)

	Active GO	Non-active GO	Total
Positive result	12	4	16
Negative result	2	5	7
Total	14	9	23

*GO* Graves’ orbitopathy, *CAS* clinical activity score

**Table 3:** Correlation between real occurrence of active or non-active GO and result of MRI (contingency table)

	Active GO	Non-active GO	Total
Positive result	13	2	15
Negative result	1	7	8
Total	14	9	23

*GO* Graves’ orbitopathy, *MRI* Magnetic resonance imaging

**Table 4:** Correlation between real occurrence of active or non-active GO and result of SPECT/CT (contingency table)

	Active GO	Non-active GO	Total
Positive result	13	1	14
Negative result	1	8	9
Total	14	9	23

*GO* Graves’ orbitopathy, *SPECT/CT* fused single photon emission computed tomography with computed tomography

It was demonstrated that of the three analysed diagnostic methods, SPECT/CT and MRI had the highest sensitivity: 0.93. Therefore, 93% of the patients with the active form of GO were positively diagnosed by means of these methods. CAS proved to have a lower sensitivity, namely 0.86.

The SPECT/CT scan was found to have the highest specificity, because as many as 89% of the patients without an active form of GO were correctly diagnosed using this method, i.e. had negative results, whereas in the case of MRI the figure proved to be 78%, while in the case of CAS - only 56%. A patient with a positive result of a SPECT/CT scan can have a 93% certainty that he or she has an active form of GO. These values are significantly lower for MRI and CAS: 87 and 75%, respectively. The SPECT/CT method seems superior also in the case of a negative result. A patient can then be 89% certain that he or she does not have an active form of GO, whereas negative results of MRI and CAS mean a certainty of 88 and 71%, respectively. It should also be added that SPECT/CT ensures the highest accuracy (i.e. probability of a correct diagnosis). A patient who undergoes a SPECT/CT scan (regardless of the obtained result) can be 91% certain of the accuracy of the diagnosis, while the same figure equals 87 and 74% in the cases of MRI and CAS, respectively (Table 5).

**Table 5:** Assessment parameters for diagnostic methods CAS, MRI and SPECT/CT in detecting active GO

	CAS	MRI	SPECT/CT	*p*
Sensitivity	0.86	0.93	0.93	0.048^a, b/c^
−95% CI	0.51	0.81	0.83	
+ 95% CI	0.92	0.98	0.99	
Specificity	0.56	0.78	0.89	0.039^a,b,c^
−95% CI	0.29	0.55	0.58	
+ 95% CI	0.56	0.89	0.99	
PPV	0.75	0.87	0.93	0.031^a,b,c^
−95% CI	0.47	0.61	0.73	
+ 95% CI	0.81	0.97	0.98	
NPV	0.71	0.88	0.89	0.049^a,b,c^
−95% CI	0.54	0.66	0.77	
+ 95% CI	0.76	0.95	0.98	
ACC	0.74	0.87	0.91	0.050^a,b,c^
−95% CI	0.63	0.58	0.92	
+ 95% CI	0.88	0.94	0.98	

*PPV* positive predictive value, NPV, negative positive predictive value, *ACC* accuracy, other abbreviations, see Table 1

^a, b, c^ - statistically significant difference between parameters of three examinations (CAS, MRI, and SPECT/CT)

^a, b/c^ - statistically significant difference between parameters of CAS and (MRI or SPECT/CT), lack of statistically significant difference between MRI and SPECT/CT

The occurrence of the active form of GO did not have an impact on the deterioration of exophthalmos or the thickness of the oculomotor muscles. The total thickness of the oculomotor muscles in one eye in the active and non-active forms of GO equalled, respectively, 40 mm +/− 6 mm and 39 mm +/− 7 mm, while the degree of exophthalmos was, respectively, 23 mm+/− 5 mm and 23 mm +/− 4.5 mm. The measurement differences were not statistically significant, *p* = 0.09 and *p* = 0.12 (Table 6).

**Table 6:** Impact of active GO on average total thickness of muscles in orbital cavity and occurrence of exophthalmos

	Average total thickness of oculomotor muscles in one eye (M, +/− SD)	Exophthalmos (M, +/− SD)	
Active GO (No 14)	40 mm +/−6 mm	23 +/− 5.0 mm	NS
Non-active GO (No 9)	39 mm +/−7 mm	23 +/− 4.5 mm	NS

GO, Graves’ orbitopathy; M, Mean; SD, standard deviation; NS, Not statistically significant; No, Number

## Discussion

The role of ^99m^Tc-DTPA scintigraphy of the orbital cavity in diagnosing the active (infiltrative-edematous) form of GO has been known for many years. The first mention of the method’s application appeared in 2002. Galuska et al. proved that ^99m^Tc-DTPA SPECT can be used to evaluate the autoimmune inflammation of the retrobulbar area in GO patients. Increased accumulation of the radiotracer in the orbital cavity, as compared to the background, indicated the presence of local inflammation. They made it clear, however, that because of the small number of examined patients (only 9), a precise evaluation of the method’s usefulness was impossible [6]. Authors from the same research institution, having conducted a study on a larger number of patients (21 persons), concluded that SPECT scintigraphy using ^99m^Tc-DTPA could be a supplementary diagnostic approach helpful in deciding whether immunosuppressive treatment should be commenced and facilitating the assessment of this treatment’s efficacy for treating GO. Like most authors, they regarded MRI as the first step in the diagnostic algorithm of GO [7]. Also Szabados writes about the usefulness of ^99m^Tc-DTPA SPECT scintigraphy in GO therapy. Authors have proved that in patients with a lack or low levels of ^99m^Tc-DTPA accumulation in the orbital cavity (in other words if there is no autoimmune inflammation or if it is mild), treatment with radiation did not bring any beneficial therapeutic effect. Therefore, a scintigraphic examination can facilitate the decision as to whether GO should be treated with systemic medications (steroids) or local methods (radiotherapy, surgery). It can also be helpful in monitoring the efficacy of treatment [2, 8].

The authors of the above studies drew their conclusions based on scintigraphic examination of orbital cavities performed using the SPECT method. This tomographic technique makes it possible to create a three-dimensional image with the use of gamma radiation emitted by a radiotracer accumulated in the organism (in this case, the orbital cavity). One advantage of this method is that it produces a spatial visualisation of the biological activity of the examined area. Speaking of biological activity in a SPECT scan of orbital cavities, we mean the detection of the presence or absence of the active form of GO (details in the introduction to this paper). A disadvantage of this technique is the relatively low resolution of the scan (> 1 cm), in comparison with other imaging methods, such as CT or MRI (where resolution < 1 mm), which makes interpretation of the result more difficult [9–11]. SPECT imaging only enables one to state whether an active form of GO is present or absent in the retro-orbital area, without allowing for a more precise indication of which of the oculomotor muscles is affected by inflammation. Besides, the presence of physiologically increased accumulation of the radiotracer in the mucous membranes of the adjacent lateral nasal sinuses can sometimes be mistakenly interpreted as increased accumulation of the radiotracer in the retro-orbital cavity, thus leading to a falsely positive result. However, our studies, aimed at evaluating the form of GO, use a hybrid method: SPECT/CT. In comparison with the traditional SPECT, the SPECT/CT technique provides a much more detailed image of the location of the accumulated radiotracer, thus improving the precision of the interpretation of the image as either physiological or pathological, depending on its exact position. This is confirmed by the very high sensitivity and specificity of the SPECT/CT method, demonstrated in our paper and equalling 93 and 89%, respectively. These values are comparable with those of MRI as far as sensitivity is concerned, and are higher as regards specificity. In our study, the sensitivity of the MRI tests was found to be 93%, whereas specificity equalled 78%. Such a high specificity of scintigraphic scans can be explained by the fact that it is capable of detecting abnormal quantities of substances at the level of picomoles, while other imaging methods (CT, MRI) can only detect millimoles [12]. In view of the above, in our study, a positive result of a SPECT/CT scan of one patient, given negative results of CAS and MRI, does not necessarily have to mean a falsely negative result, as was assumed for the sake of the statistical analysis (Tables 1 and 2). The patient can simply have the active form of GO of low severity, impossible to detect with CAS and MRI. On the other hand, a single falsely negative result can result from the difficulty of assessing the accumulation of the radiotracer in the medial oculomotor muscles due to the presence of physiologically increased build-up of the radiotracer in the adjacent mucosa of the nasal sinuses. However, the number of patients we have examined is too low to allow us to state this with absolute certainty.

What is also interesting is that our study suggests a lack of statistically significant difference between the active and non-active forms of GO taking into consideration the severity of exophthalmos and the thickness of the oculomotor muscles. This can be explained by the relatively small group of investigated patients, but also by the fact that no norms exist for the width of muscles in the active or non-active forms of GO. Frequently, the muscles of a patient with active GO are smaller in volume than those of a patient with non-active GO, and vice versa. It is similar with the severity of exophthalmos. In our study, this is reflected in the low specificity of CAS in diagnosing the active form of GO, equalling just 56%. The reason is that in order to differentiate the forms of GO, the method bases on the evaluation of clinical symptoms manifesting mainly as an expansion of the oculomotor muscles and the occurrence of exophthalmos.

Finally, it should be emphasised that the SPECT/CT examination using ^99m^Tc-DTPA is safe in terms of radiation protection. The overall dose of ionising radiation absorbed by the patient is the sum of: 1mSV received during the SPECT scan and about 0.6 mSV received during the low-dose CT. It is, therefore, only a small fraction of the absorbed dose of ionising radiation which is universally regarded as safe for healthy persons who work with ionising radiation, i.e. 20mSV per year [13, 14]. Moreover, taking into account the economic aspects, it should be noted that the method is less expensive than MRI, the standard investigation in this condition.

## Conclusion

On the basis of our analysis, it can be concluded that the ^99m^Tc-DTPA SPECT/CT method provides a very useful tool for evaluating the active form of GO, in many respects superior to the routinely applied methods. It seems that it can be used as a referential diagnostic technique in GO, along with MRI.

## Abbreviations

^99m^Tc-DTPA: Diethylenetriaminepentaacetic acid tagged with ^99m^Tc
ACC: Accuracy
CAS: Clinical Activity Score
CT: Computed tomography
GO: Graves’ orbithopathy
MRI: Magnetic resonance imaging
NPV: Negative predictive value
PPV: Positive predictive value
SPECT: Single-photon emission computed tomography
SPECT/CT: SPECT examination fused with a CT scan
TRAb: TSH receptor antibody
TSH: Thyroid-stimulating hormone
USG: Ultrasonography
